# Supplementary material for: The Effects of Optimal Dietary Vitamin D3 on Growth and Carcass Performance, Tibia Traits, Meat Quality, and Intestinal Morphology of Chinese Yellow-Feathered Broiler Chickens
Source: Animals (Basel). 2024 Mar 16;14(6):920. doi: 10.3390/ani14060920 (PMC10967611; doi:10.3390/ani14060920)
Supplement: Supplementary file 1 [file animals-14-00920-s001.zip › Figure S1-2.pdf]

Supplementary Figure

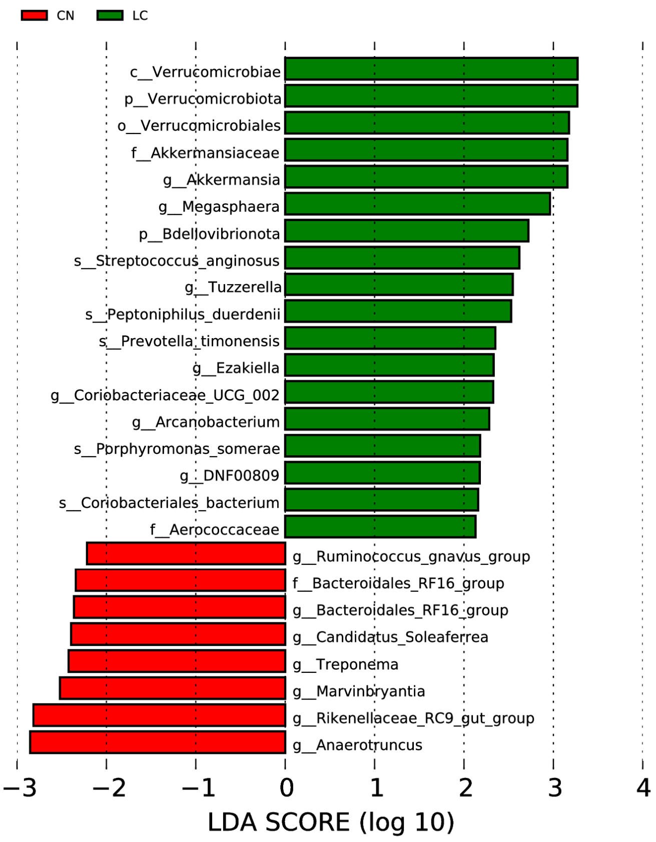

Figure S1. Comparison of the classification of the cecal microbiota of broilers between control (CN) and low concentration (LC) groups using the linear discriminant analysis (LDA) effect size (LefSe) method

Note: The LDA value distribution histogram shows the species with significant differences in abundance between the two groups, and the length of the histogram represents the impact of different species.

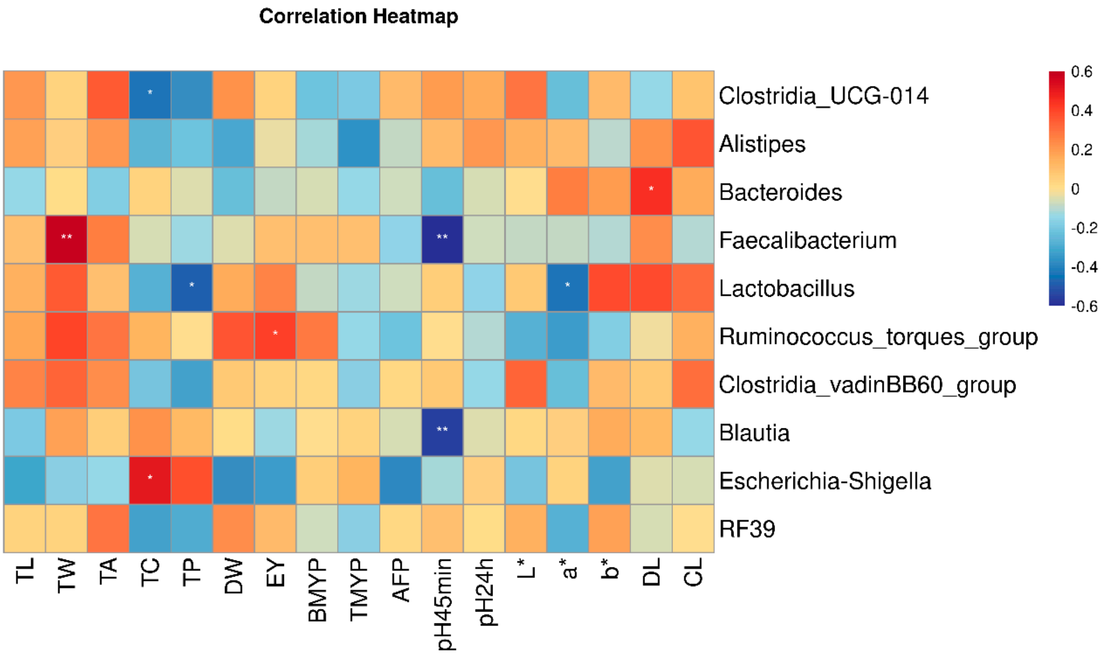

Figure S2. Pearson correlation analysis of the top 10 contributed genera with the phenotype characteristics of broilers at 63 days.

Note: * indicates a significant difference at $P < 0.05$, and ** indicates a significant difference at $P < 0.01$. TL, total lipids; TW, total weight; TA, total albumin; TC, total cholesterol; TP, total protein; DW, dressed weight; EY, eviscerated yield; BMYP, breast muscle yield percentage; TMYP, thigh muscle yield percentage; AFP, abdominal fat percentage; pH45min, initial pH; pH24h, pH at 24 h; L*, lightness; a*, redness; b*, yellowness; DL, CL.
