# Supplementary material for: The Effects of Optimal Dietary Vitamin D3 on Growth and Carcass Performance, Tibia Traits, Meat Quality, and Intestinal Morphology of Chinese Yellow-Feathered Broiler Chickens
Source: Animals (Basel). 2024 Mar 16;14(6):920. doi: 10.3390/ani14060920 (PMC10967611; doi:10.3390/ani14060920)
Supplement: Supplementary file 1 [file animals-14-00920-s001.zip › Table S1-5.pdf]

Table S1. Basic diet composition and nutrient level for yellow-feathered broilers

Item	1 to 21 d	22 to 42 d	43 to 63 d
**Ingredients**	601.6	448.1	481.2
Corn	/	200.0	200.0
Barley	238.8	137.9	66.40
Soybean meal	40.0	60.0	80.0
Cotton pulp	36.45	49.67	60.00
Corn gluten powder	30.00	20.00	20.00
Flour	9.90	40.00	50.10
Grade IV soybean oil	12.60	9.90	8.20
Calcium hydrogen Phosphate	13.20	13.70	13.80
Stone powder	2.40	2.50	2.50
Sodium chloride	1.50	1.50	1.50
Baking soda	1.00	1.00	1.00
Anti-mildew agent	4.80	4.80	4.80
Premix	5.04	7.06	7.60
Lysine	1.76	2.57	1.56
Solid methionine	0.88	1.39	1.38
Threonine	1000	1000	1000
**Nutritional level**			
Moisture (%)	12.96	12.43	12.34
Crude protein (%)	20.50	18.50	17.00
Crude fat (%)	3.54	6.30	7.41
Crude fiber (%)	2.40	2.91	2.89
Calcium (%)	0.90	0.85	0.80
Phosphorus (%)	0.60	0.55	0.50
Sodium (%)	0.16	0.16	0.16
Potassium (%)	0.73	0.62	0.51
Electrolyte balance (mEq/kg)	203	167	139
Metabolizable energy (MJ/kg)	12.14	12.77	13.19
Lysine (%)	1.20	1.10	1.00

Note: Per kg diet: Vitamin A (VA) 1000 IU, Vitamin D_3 (VD_3) 0 IU, Vitamin E (VE) 16 IU, Vitamin K (VK) 2.0 mg, Vitamin B_1 (VB_1) 2.0 mg, Vitamin B_2 (VB_2) 6.4 mg, Vitamin B_6 (VB_6) 2.0 mg, Vitamin B_{12} (VB_{12}) 0.012 mg, calcium pantothenate 10.0 mg, nicotinamide 26.0 mg, folate 1.0 mg, biotin 0.1 mg, copper 8 mg, choline 700 mg, zinc 60 mg, manganese 80 mg, iron 80 mg, iodine 0.35 mg, selenium 0.15 mg.

Table S2. Effects of dietary supplementation with different levels of VD_3 on the serum biochemical indices of broilers

Parameter	Groups			
	CN	LC	MC	HC
Total protein (g/L)	40.59 ± 1.36	40.35 ± 1.22	39.26 ± 0.42	37.81 ± 1.42
Albumin (g/L)	14.24 ± 0.48	14.86 ± 0.51	14.26 ± 0.19	13.74 ± 0.45
Globulin (g/L)	26.35 ± 0.97	25.49 ± 0.86	24.87 ± 0.85	24.90 ± 0.89
Glucose (mmol/L)	7.64 ± 0.54	9.34 ± 0.68	8.59 ± 0.95	8.83 ± 0.65
AST (U/L)	258.40 ± 8.40	244.10 ± 6.95	253.56 ± 8.83	254.67 ± 4.04
Creatinine (μmol/L)	9.57 ± 0.72	9.00 ± 0.52	8.50 ± 0.57	8.44 ± 0.50
Urea (mmol/L)	0.51 ± 0.03	0.43 ± 0.04	0.43 ± 0.04	0.43 ± 0.03
Uric acid (μmol/L)	191.33 ± 15.34	171.60 ± 12.28	188.30 ± 7.04	169.60 ± 19.87
Total cholesterol (mmol/L)	3.86 ± 0.19	3.91 ± 0.13	3.74 ± 0.15	3.82 ± 0.11
HDL-C (mmol/L)	2.42 ± 0.11	2.32 ± 0.09	2.41 ± 0.07	2.39 ± 0.09
LDL-C (mmol/L)	1.09 ± 0.08	0.97 ± 0.05	0.96 ± 0.08	0.98 ± 0.04
Triglyceride (mmol/L)	0.60 ± 0.05	0.51 ± 0.03	0.50 ± 0.04	0.50 ± 0.05

Note: The values were calculated as the means ± standard error of the mean (n = 12); CN, control; LC, low concentration of VD_3; MC, medium concentration of VD_3; HC, high concentration of VD_3; AST, aspartate aminotransferase; HDL-C, high-density lipoprotein cholesterol; LDL-C, low-density lipoprotein cholesterol.

Table S3. Effects of dietary supplementation with different levels of VD_3 on the serum calcium and phosphorus contents of broilers

Parameter	Groups			
	CN	LC	MC	HC
Ca (mmol/L)				
21 d	1.11 ± 0.03^{b}	1.09 ± 0.01^{b}	1.20 ± 0.01^{a}	1.21 ± 0.02^{a}
42 d	1.17 ± 0.01	1.16 ± 0.02	1.17 ± 0.02	1.19 ± 0.02
63 d	1.52 ± 0.02	1.54 ± 0.02	1.51 ± 0.02	1.53 ± 0.02
P (mmol/L)				
21 d	2.04 ± 0.08	2.10 ± 0.05	2.10 ± 0.05	1.94 ± 0.10
42 d	1.96 ± 0.05^{b}	2.10 ± 0.04^{b}	1.99 ± 0.05^{b}	2.31 ± 0.06^{a}
63 d	2.34 ± 0.14	2.55 ± 0.09	2.49 ± 0.10	2.43 ± 0.07

Note: The values were calculated as the means ± standard error of the mean (n = 12); a and b denote values that differ significantly at $P < 0.05$. CN, control; LC, low concentration of VD_3; MC, medium concentration of VD_3; HC, high concentration of VD_3.

Table S4. Effects of dietary supplementation with different levels of VD_3 on the carcass traits of broilers

Parameter	Groups			
	CN	LC	MC	HC
LW (g)	1760.17 ± 19.42^{b}	2031.67 ± 20.52^{a}	1990.83 ± 14.11^{a}	2022.50 ± 21.32^{a}
DW (g)	1608.84 ± 21.29^{b}	1869.47 ± 19.09^{a}	1821.81 ± 14.23^{a}	1867.48 ± 19.81^{a}
EY (g)	1213.09 ± 21.74^{b}	1447.67 ± 18.37^{a}	1410.68 ± 12.52^{a}	1451.28 ± 16.46^{a}
BMYP (%)	12.48 ± 0.38^{b}	14.22 ± 0.45^{a}	14.35 ± 0.34^{a}	14.28 ± 0.34^{a}
TMYP (%)	20.78 ± 0.16	20.71 ± 0.53	20.31 ± 0.40	19.97 ± 0.23
AFP (%)	3.71 ± 0.33^{a}	2.66 ± 0.18^{b}	3.43 ± 0.17^{ab}	3.37 ± 0.23^{ab}

Note: The values were calculated as the means ± standard error of the mean (n = 12); a and b denote values that differ significantly at $P < 0.05$. CN, control; LC, low concentration of VD_3; MC, medium concentration of VD_3; HC, high concentration of VD_3; LW, live weight at slaughter; DW, dressed weight; EY, eviscerated yield; BMYP, breast muscle yield percentage; TMYP, thigh muscle yield percentage; AFP, abdominal fat percentage.

Table S5. Effects of dietary supplementation with different levels of VD_3 on the meat quality of broilers

Parameter	Groups			
	CN	LC	MC	HC
Breast muscle				
PH_{45min}	5.73 ± 0.08^{ab}	5.67 ± 0.10^{b}	5.83 ± 0.11^{ab}	5.96 ± 0.06^{a}
PH_{24h}	5.10 ± 0.10	5.24 ± 0.09	5.03 ± 0.05	5.01 ± 0.06
L*	53.21 ± 0.97	50.93 ± 1.27	53.31 ± 0.75	51.91 ± 1.37
a*	8.27 ± 0.27	7.97 ± 0.39	8.11 ± 0.40	8.58 ± 0.24
b*	14.84 ± 0.68^{a}	12.69 ± 0.55^{b}	13.13 ± 0.37^{b}	12.04 ± 0.33^{b}
Drip loss (%)	2.48 ± 0.09	2.23 ± 0.11	2.32 ± 0.12	2.41 ± 0.12
Cooking loss (%)	5.73 ± 0.08^{ab}	5.67 ± 0.10^{b}	5.83 ± 0.11^{ab}	5.96 ± 0.06^{a}
Thigh muscle				
PH_{45min}	5.96 ± 0.09^{ab}	5.86 ± 0.03^{b}	5.94 ± 0.07^{ab}	6.07 ± 0.05^{a}
PH_{24h}	5.68 ± 0.06	5.68 ± 0.04	5.65 ± 0.05	5.70 ± 0.06
L*	51.01 ± 1.76	51.81 ± 1.91	53.35 ± 1.86	51.71 ± 1.33
a*	17.40 ± 1.20	17.53 ± 1.51	17.39 ± 0.92	17.03 ± 1.09
b*	13.09 ± 0.71	11.36 ± 0.88	11.99 ± 0.50	11.81 ± 0.69
Drip loss (%)	2.18 ± 0.07	1.89 ± 0.14	2.04 ± 0.10	2.07 ± 0.13
Cooking loss (%)	5.96 ± 0.09^{ab}	5.86 ± 0.03^{b}	5.94 ± 0.07^{ab}	6.07 ± 0.05^{a}

Note: The values were calculated as the means ± standard error of the mean (n = 12); a and b denote values that differ significantly at $P < 0.05$. CN, control; LC, low concentration of VD_3; MC, medium concentration of VD_3; HC, high concentration of VD_3; L*, lightness; a*, redness; b*, yellowness.

Table S6A. Quality control and preprocessing of metagenomic datasets
Raw_tags, original sequencing; Base, the total base number of the effective tags; Q20, the percentage of the effective tags with the Q value of each base ≥ 20 (sequencing error rate ≤ 1%); Q30, the percentage of effective tags with the Q value of each base ≥ 30 (sequencing error rate ≤ 0.1%); GC%, the percentage of GC bases in the effective tags.
Table S6B. The compositions of the top 10 genera in the cecal contents of broilers
Table S6C. The alpha diversity of the cecal microbiotas in control (CN) and low concentration (LC) groups
Chao1: The number of estimated species in the tested samples. The more low-abundance species in the community, the higher the Chao1 index;
Goods_coverage: The higher the sequencing coverage, the greater the index;
Observed_OTUs: the number of species observed intuitively (the larger index, the more species observed); Shannon: The total number and proportion of classifications in the sample. The higher the community diversity, the more uniform the species distribution, and the larger the Shannon index; Simpson: The diversity and evenness of species distribution. The better the species evenness, the higher the Simpson index.
Table 6D. The microorganisms with significant differences at the genus level

Table S7A. Sequencing data quality assessment
Table S7B. Mapping results with reference genomes
Table S7C. Mapping reference area statistics
Table S7D. Liver transcriptome analysis between the control (CN) and low concentration (LC) groups
Table S7E. The significantly expressed genes between the control (CN) and low concentration (LC) groups
Table S7F. Kyoto Encyclopedia of Genes and Genomes (KEGG) analysis of differentially expressed genes
Table S7G. Gene set enrichment analysis (GSEA) analysis of expressed genes
